# Supplementary material for: New Meroterpenes from South China Sea Soft Coral Litophyton brassicum
Source: Mar Drugs. 2024 Aug 30;22(9):392. doi: 10.3390/md22090392 (PMC11433555; doi:10.3390/md22090392)
Supplement: Supplementary file 1 [file marinedrugs-22-00392-s001.zip › marinedrugs-3161260-supplementary.pdf]

Supporting Information

Figure S1 HRESIMS spectrum of compound **1** ... 2
Figure S2 ^{1}H NMR spectrum of compound **1** in $CDCl_3$, 600 MHz ... 2
Figure S3 ^{13}C NMR spectrum of compound **1** in $CDCl_3$, 150 MHz ... 3
Figure S4 DEPT 135 spectrum of **1** in $CDCl_3$, 150 MHz ... 3
Figure S5 ^{1}H-^{1}H COSY spectrum of **1** in $CDCl_3$, 600 MHz ... 4
Figure S6 HSQC spectrum of **1** in $CDCl_3$, 150 MHz ... 4
Figure S7 HMBC spectrum of **1** in $CDCl_3$, 150 MHz ... 5
Figure S8 1D NOE spectrum of **1** in $CDCl_3$, 600 MHz (H-6) ... 5
Figure S9 HRESIMS spectrum of compound **2** ... 6
Figure S10 ^{1}H NMR spectrum of compound **2** in $CDCl_3$, 600 MHz ... 6
Figure S11 ^{13}C NMR spectrum of compound **2** in $CDCl_3$, 150 MHz ... 7
Figure S12 DEPT 135 spectrum of **2** in $CDCl_3$, 150 MHz ... 7
Figure S13 ^{1}H-^{1}H COSY spectrum of **2** in $CDCl_3$, 600 MHz ... 8
Figure S14 HSQC spectrum of **2** in $CDCl_3$, 150 MHz ... 8
Figure S15 HMBC spectrum of **2** in $CDCl_3$, 150 MHz ... 9
Figure S16 1D NOE spectrum of **2** in $CDCl_3$, 600 MHz (H-6) ... 9
Figure S17 1D NOE spectrum of **2** in $CDCl_3$, 600 MHz (H-10) ... 10
Figure S18 HRESIMS spectrum of compound **3** ... 10
Figure S19 ^{1}H NMR spectrum of compound **3** in $CDCl_3$, 600 MHz ... 11
Figure S20 ^{13}C NMR spectrum of compound **3** in $CDCl_3$, 150 MHz ... 11
Figure S21 DEPT 135 spectrum of **3** in $CDCl_3$, 150 MHz ... 12
Figure S22 ^{1}H-^{1}H COSY spectrum of **3** in $CDCl_3$, 600 MHz ... 12
Figure S23 HSQC spectrum of **3** in $CDCl_3$, 150 MHz ... 13
Figure S24 HMBC spectrum of **3** in $CDCl_3$, 150 MHz ... 13
Figure S25 1D NOE spectrum of **3** in $CDCl_3$, 600 MHz (H-6) ... 14
Figure S26 1D NOE spectrum of **3** in $CDCl_3$, 600 MHz (H-10) ... 14
Figure S27 HRESIMS spectrum of compound **4** ... 15
Figure S28 ^{1}H NMR spectrum of compound **4** in $CDCl_3$, 600 MHz ... 15
Figure S29 ^{13}C NMR spectrum of compound **4** in $CDCl_3$, 150 MHz ... 16
Figure S30 DEPT 135 spectrum of **4** in $CDCl_3$, 150 MHz ... 16
Figure S31 ^{1}H-^{1}H COSY spectrum of **4** in $CDCl_3$, 600 MHz ... 17
Figure S32 HSQC spectrum of **4** in $CDCl_3$, 150 MHz ... 17
Figure S33 HMBC spectrum of **4** in $CDCl_3$, 150 MHz ... 18
Figure S34 1D NOE spectrum of **4** in $CDCl_3$, 600 MHz (H-6) ... 18
Figure S35 1D NOE spectrum of **4** in $CDCl_3$, 600 MHz (H-10) ... 19
Figure S36 The experimental ECD spectrum of **1** ... 19
Figure S37 The experimental ECD spectrum of **2** ... 20

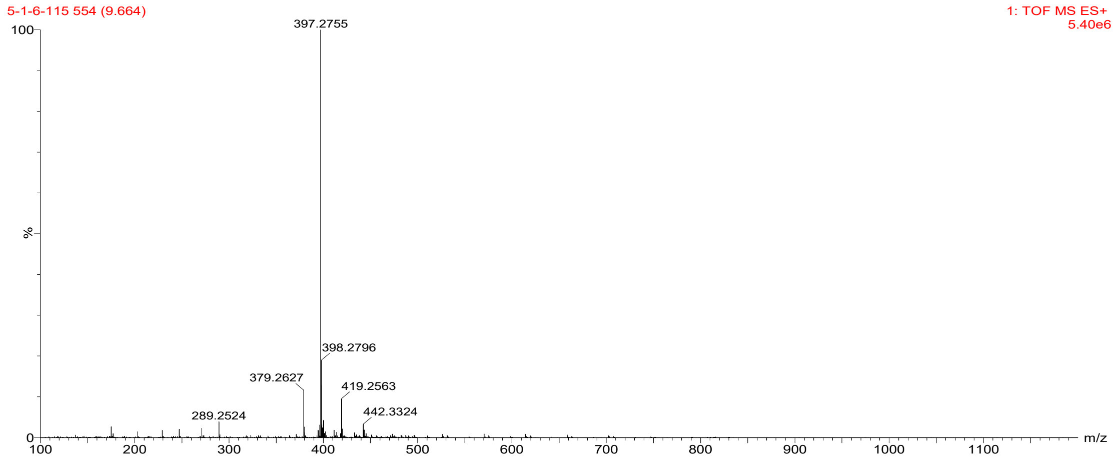

Figure S1 HRESIMS spectrum of compound **1**

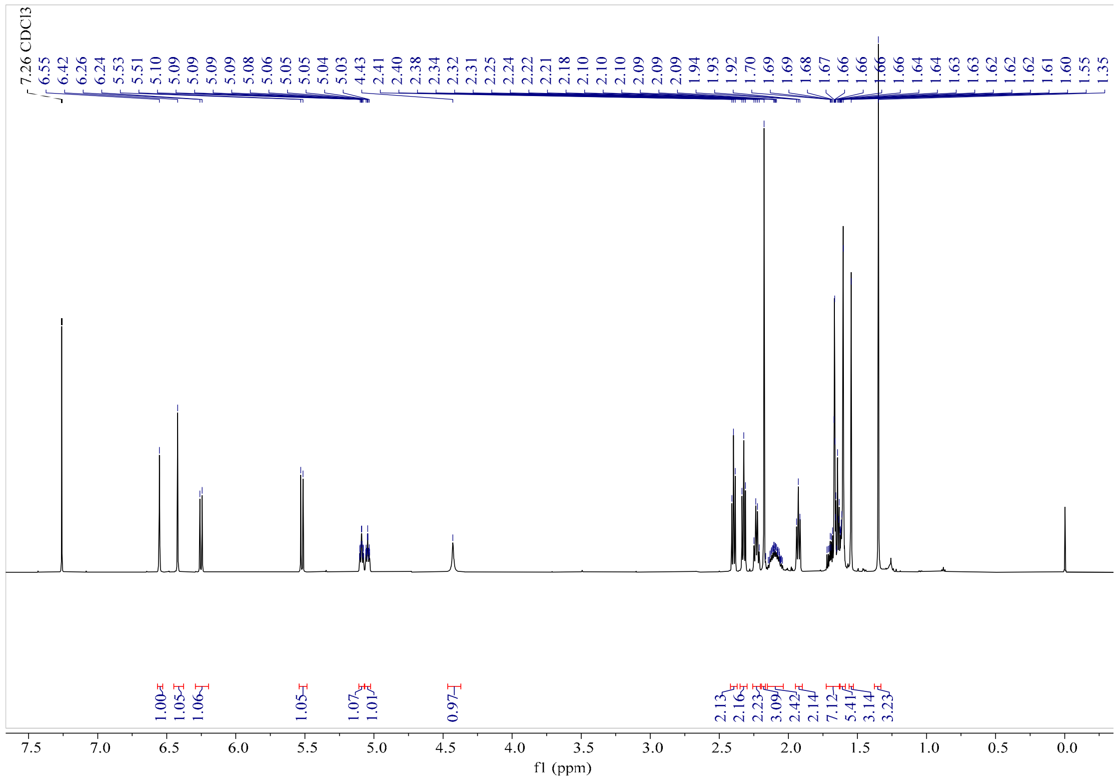

Figure S2 ^{1}H NMR spectrum of compound **1** in $CDCl_3$, 600 MHz

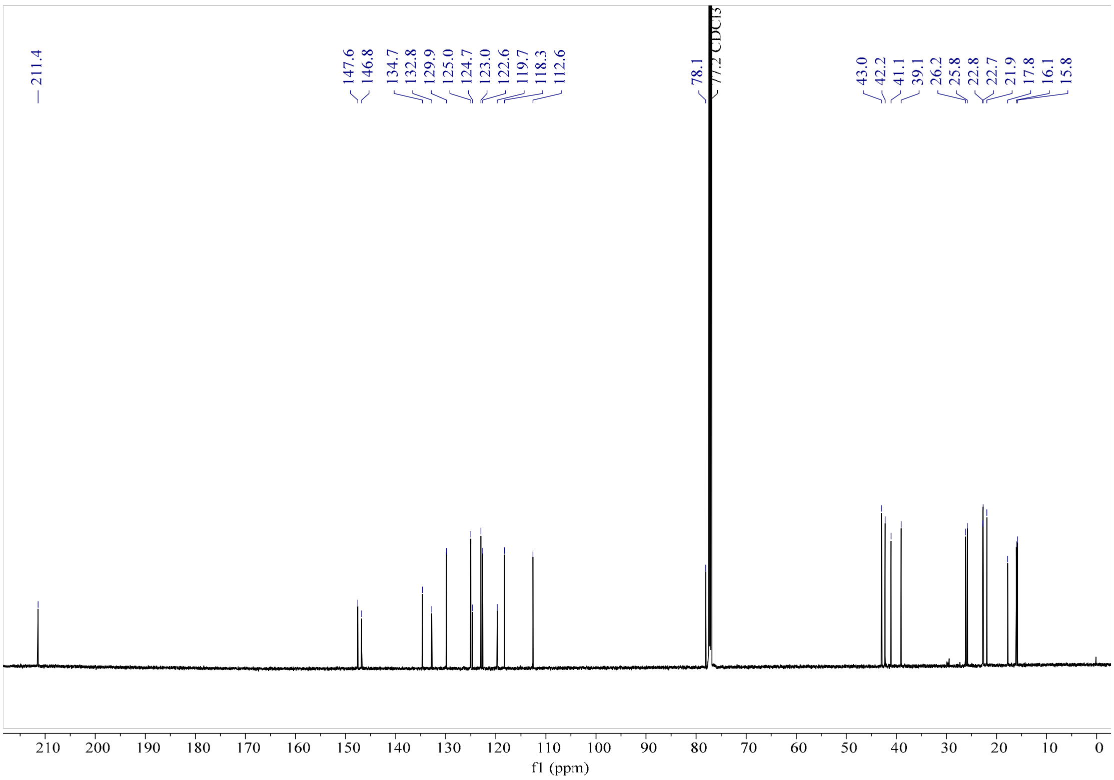

Figure S3 ^{13}C NMR spectrum of compound **1** in $CDCl_3$, 150 MHz

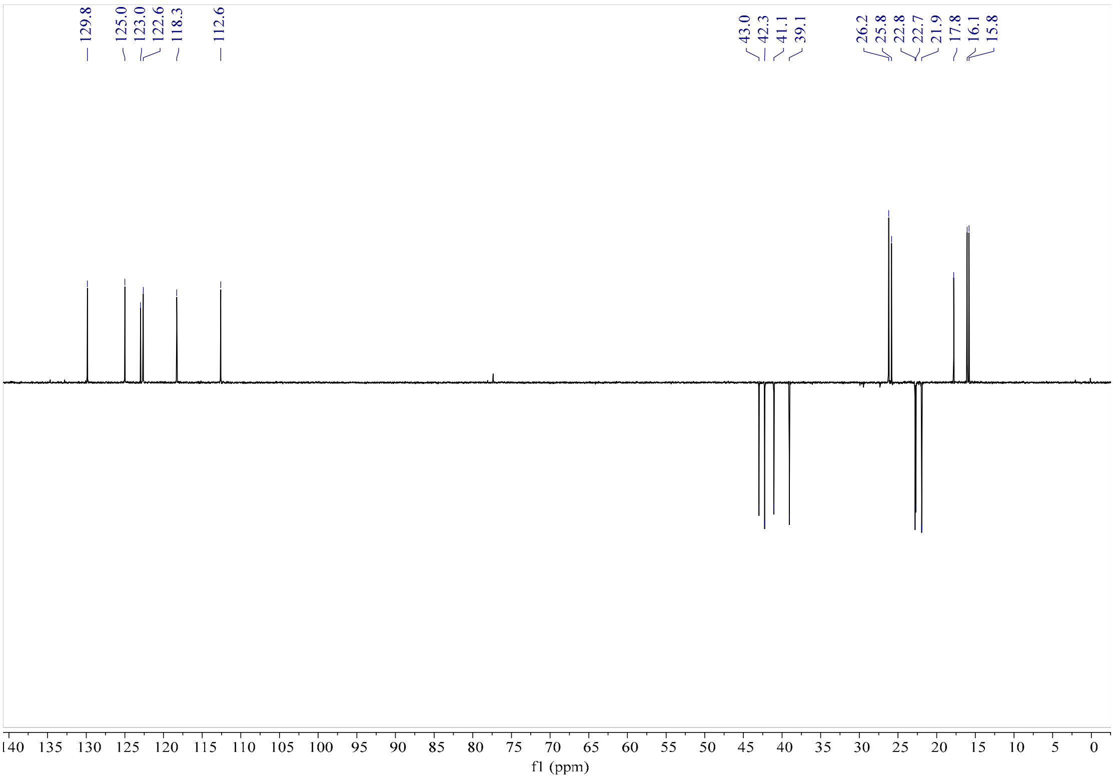

Figure S4 DEPT 135 spectrum of **1** in $CDCl_3$, 150 MHz

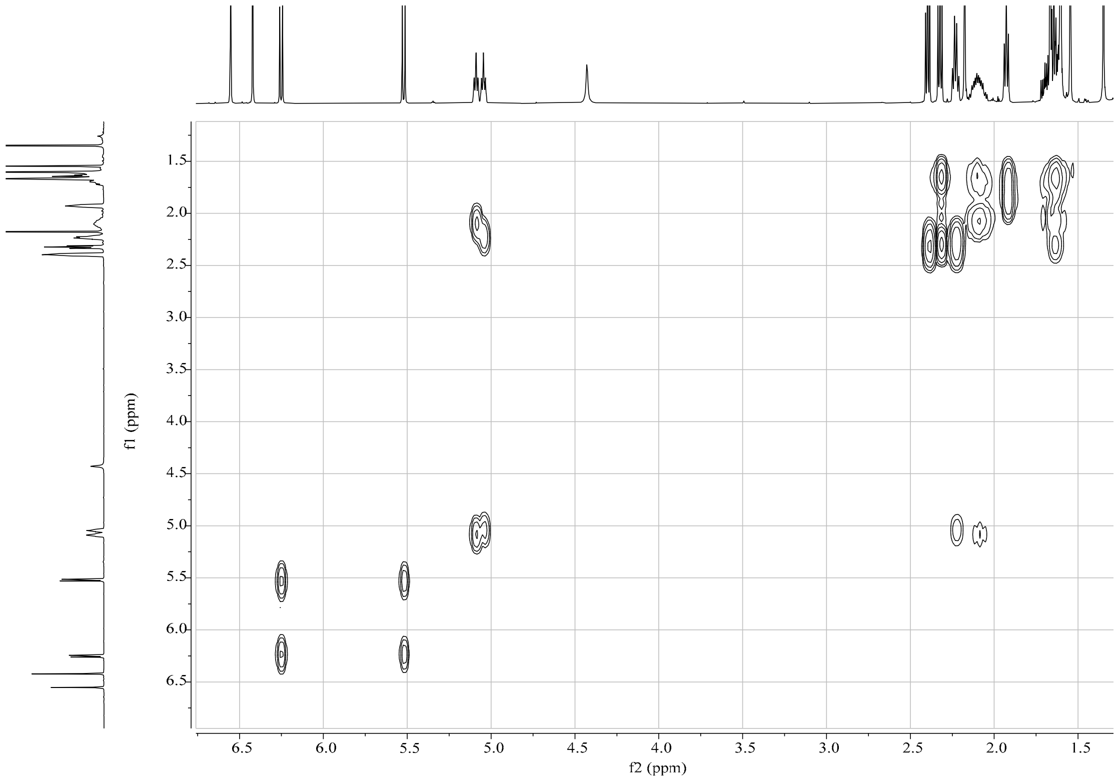

Figure S5 ^{1}H-^{1}H COSY spectrum of **1** in $CDCl_3$, 600 MHz

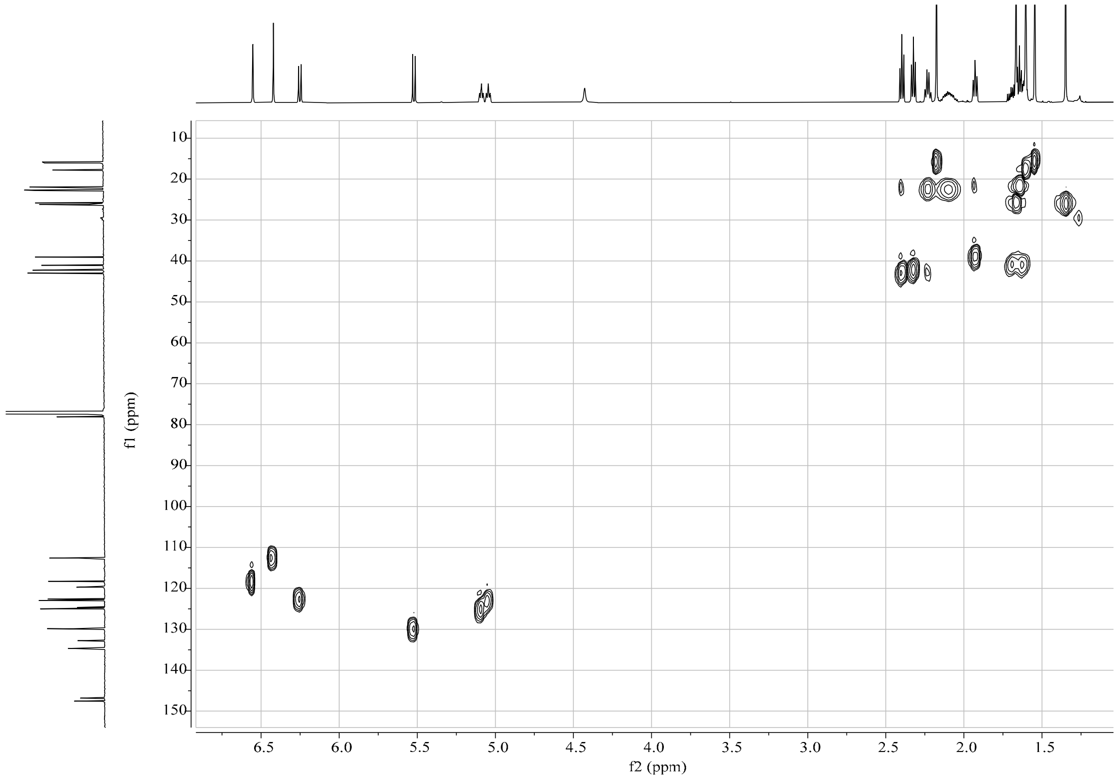

Figure S6 HSQC spectrum of **1** in $CDCl_3$, 150 MHz

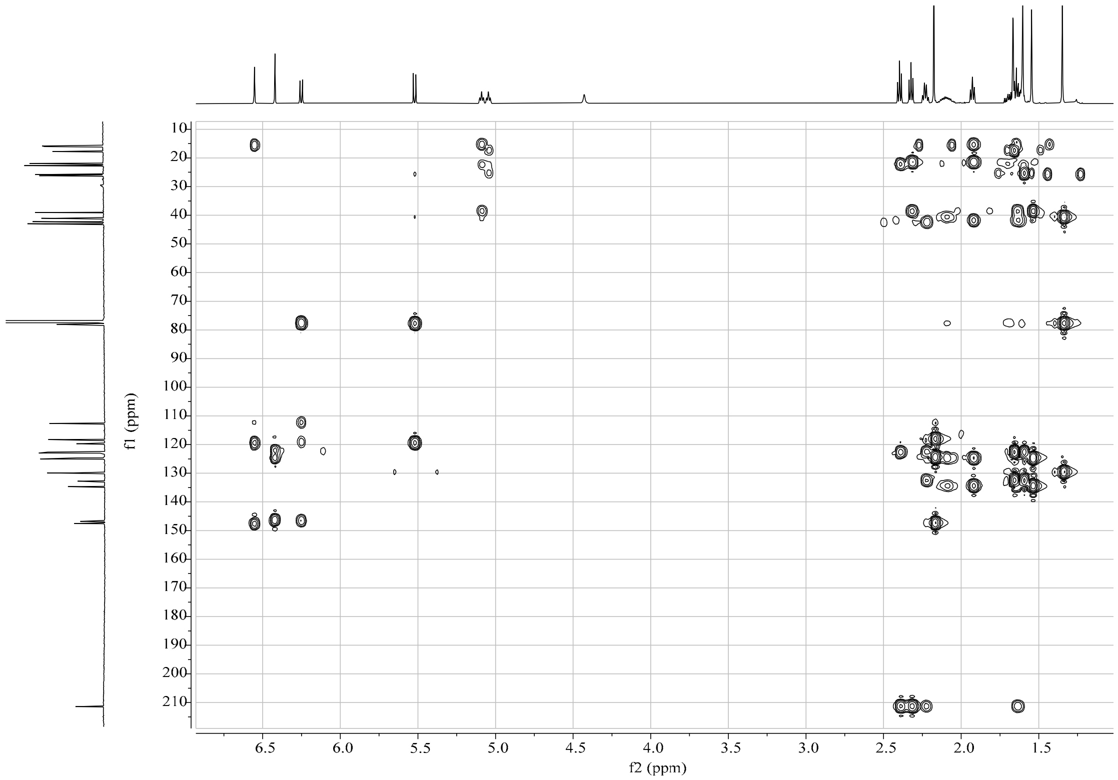

Figure S7 HMBC spectrum of **1** in $CDCl_3$, 150 MHz

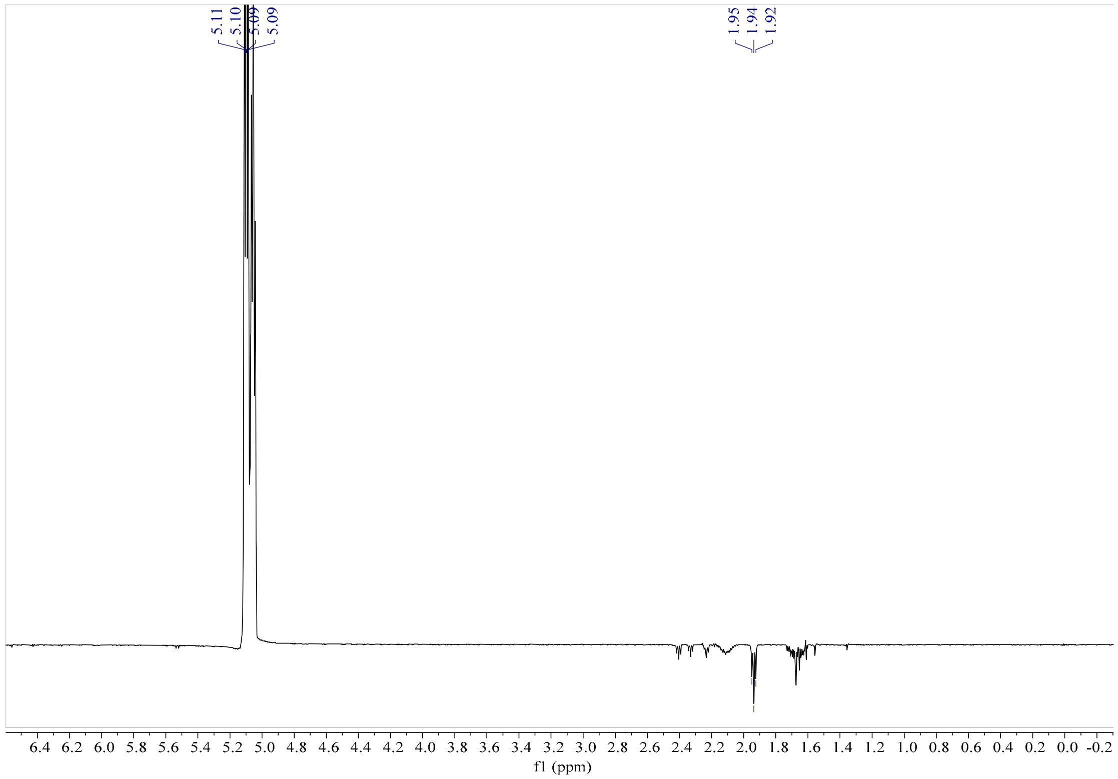

Figure S8 1D NOE spectrum of **1** in $CDCl_3$, 600 MHz (H-6)

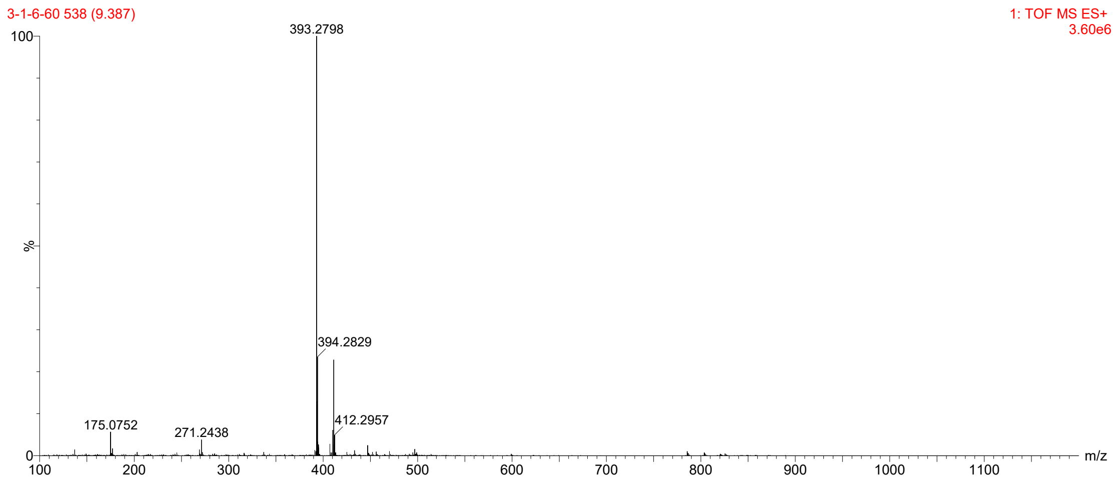

Figure S9 HRESIMS spectrum of compound **2**

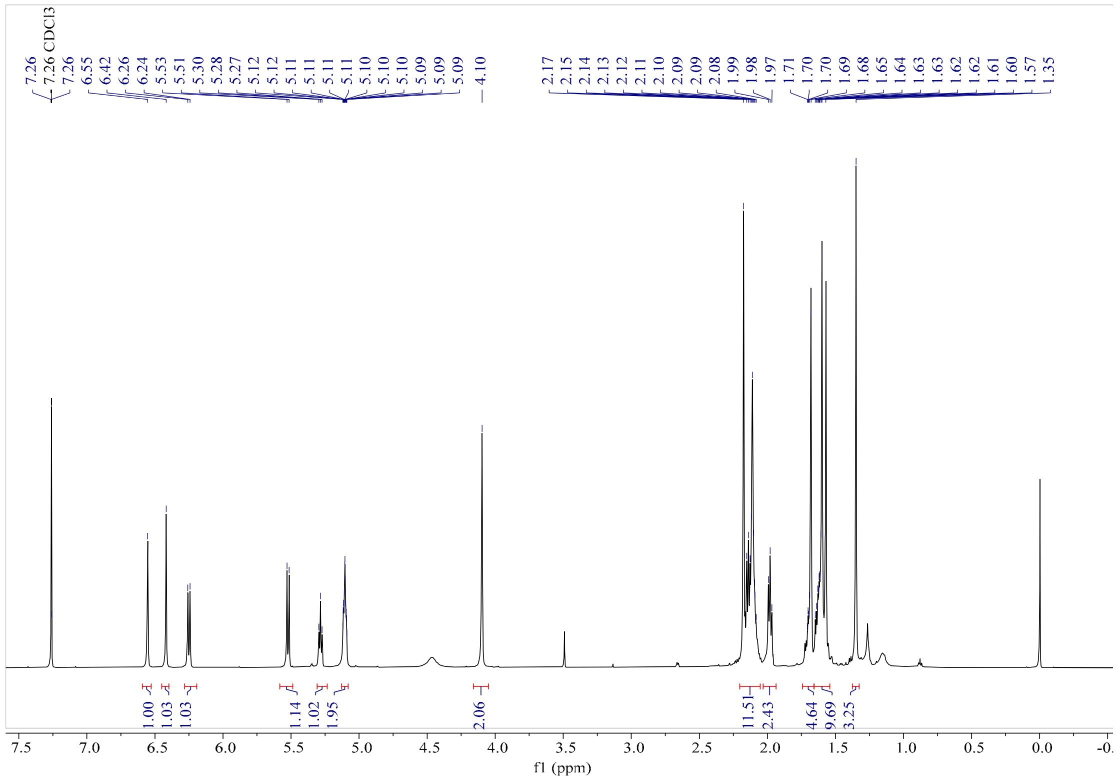

Figure S10 ^{1}H NMR spectrum of compound **2** in $CDCl_3$, 600 MHz

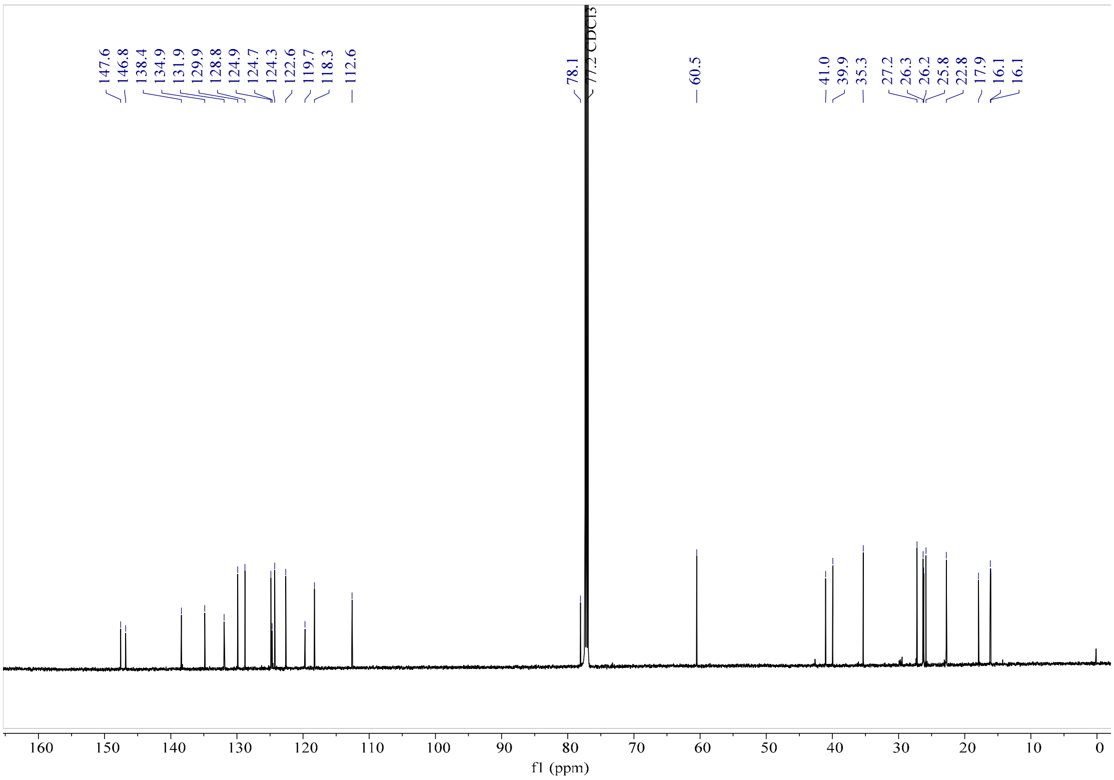

Figure S11 ^{13}C NMR spectrum of compound **2** in $CDCl_3$, 150 MHz

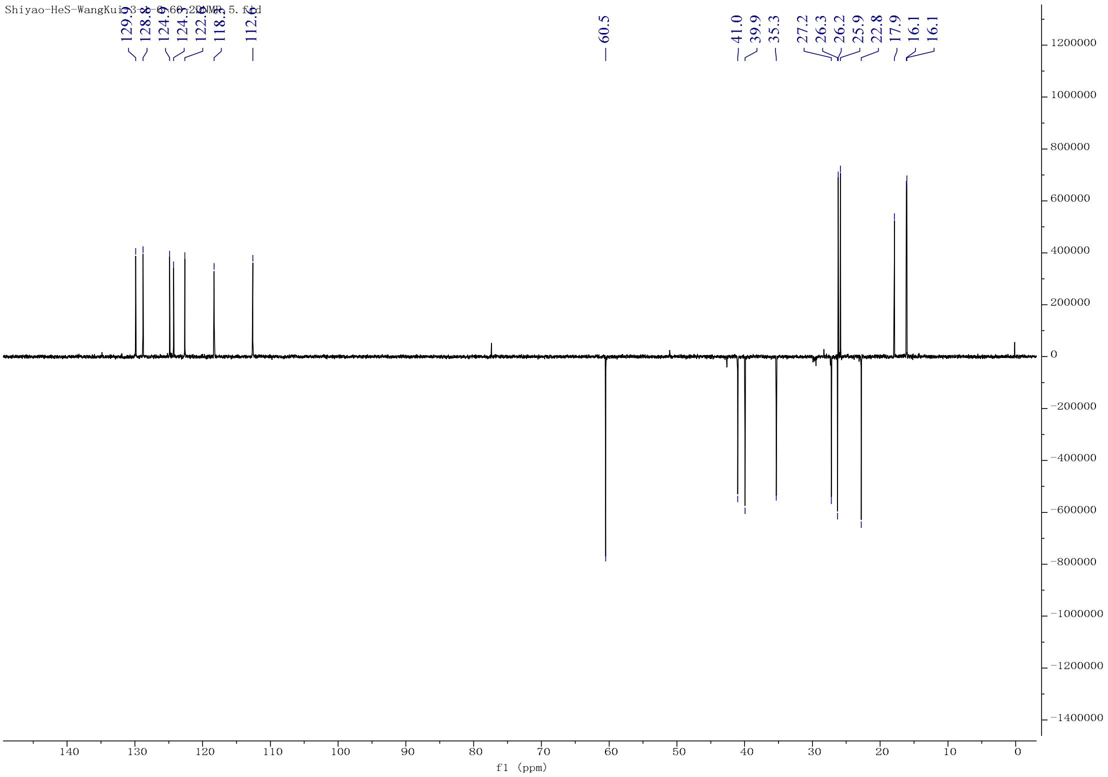

Figure S12 DEPT 135 spectrum of **2** in $CDCl_3$, 150 MHz

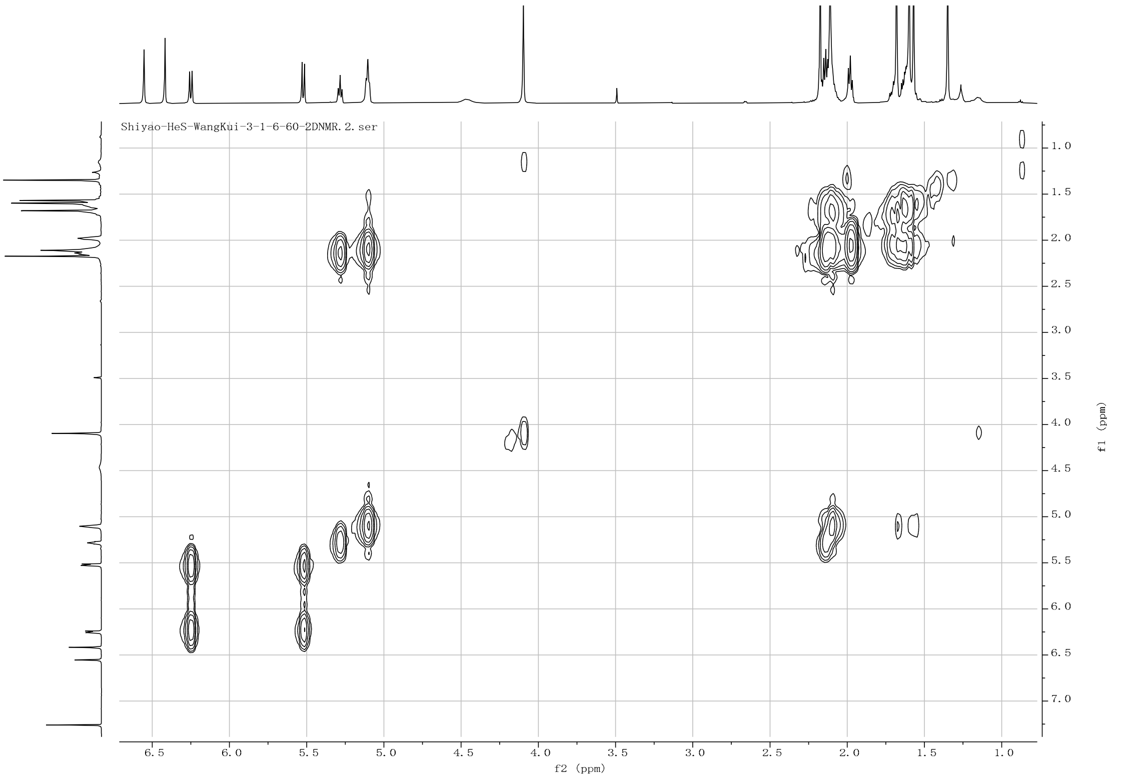

Figure S13 ^{1}H-^{1}H COSY spectrum of **2** in $CDCl_3$, 600 MHz

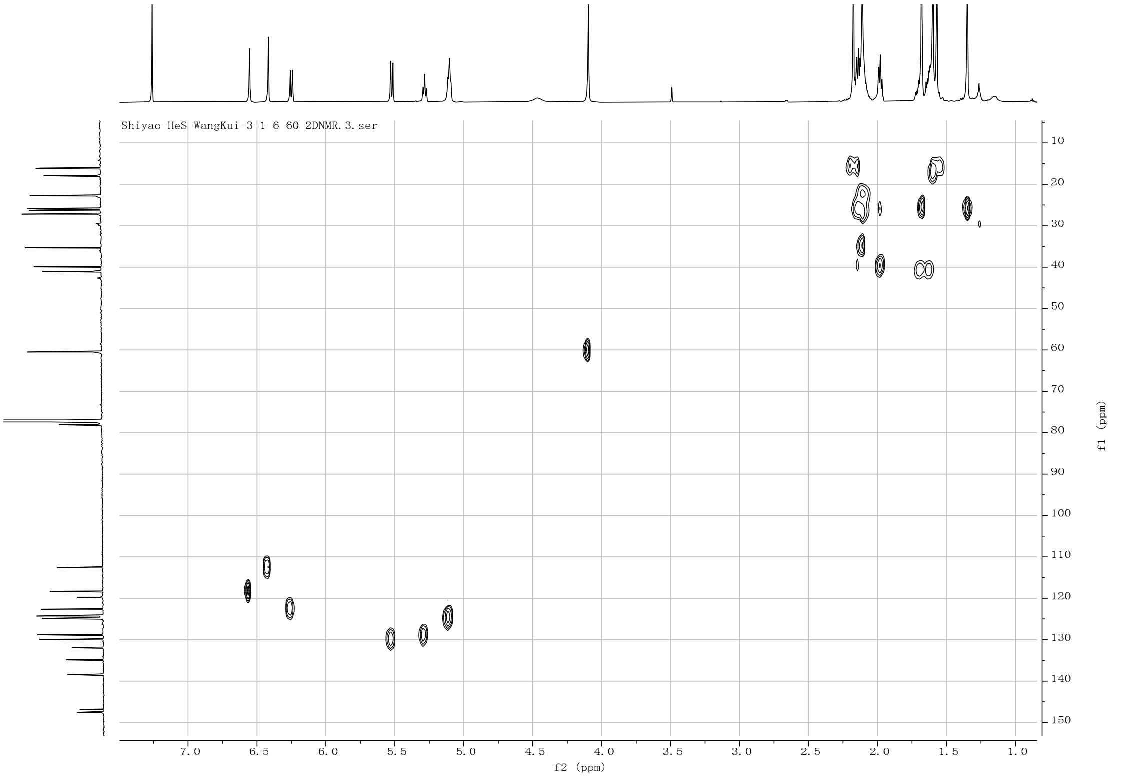

Figure S14 HSQC spectrum of **2** in $CDCl_3$, 150 MHz

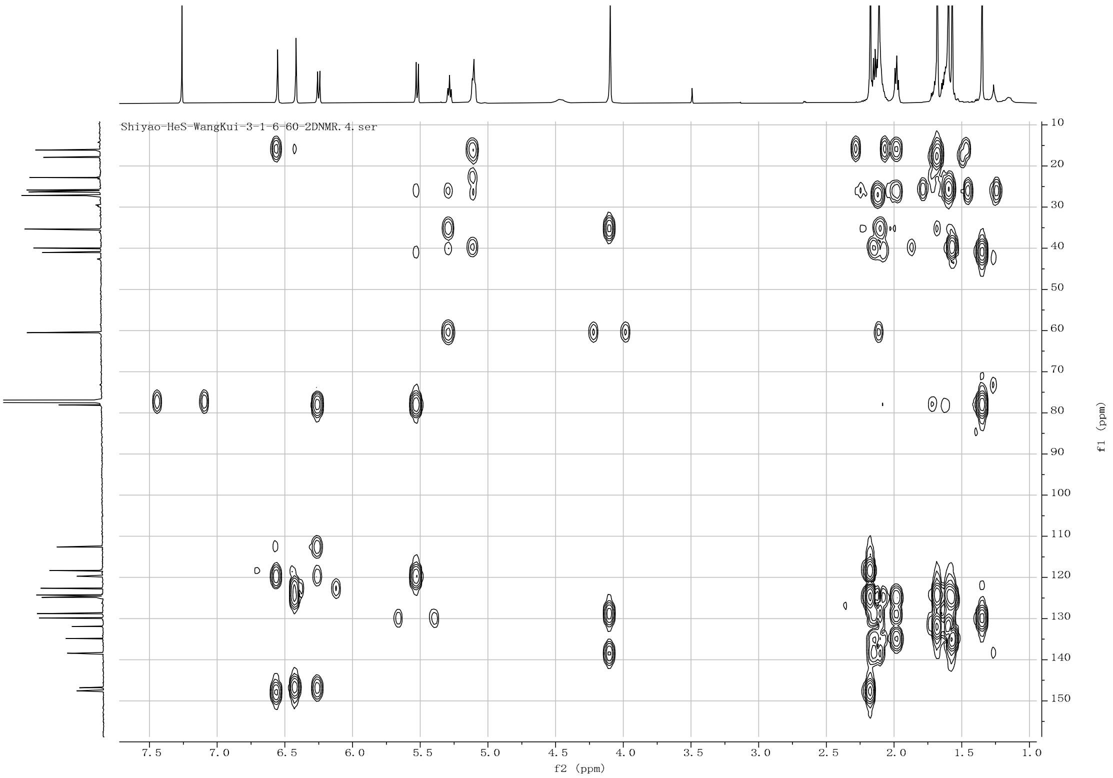

Figure S15 HMBC spectrum of **2** in $CDCl_3$, 150 MHz

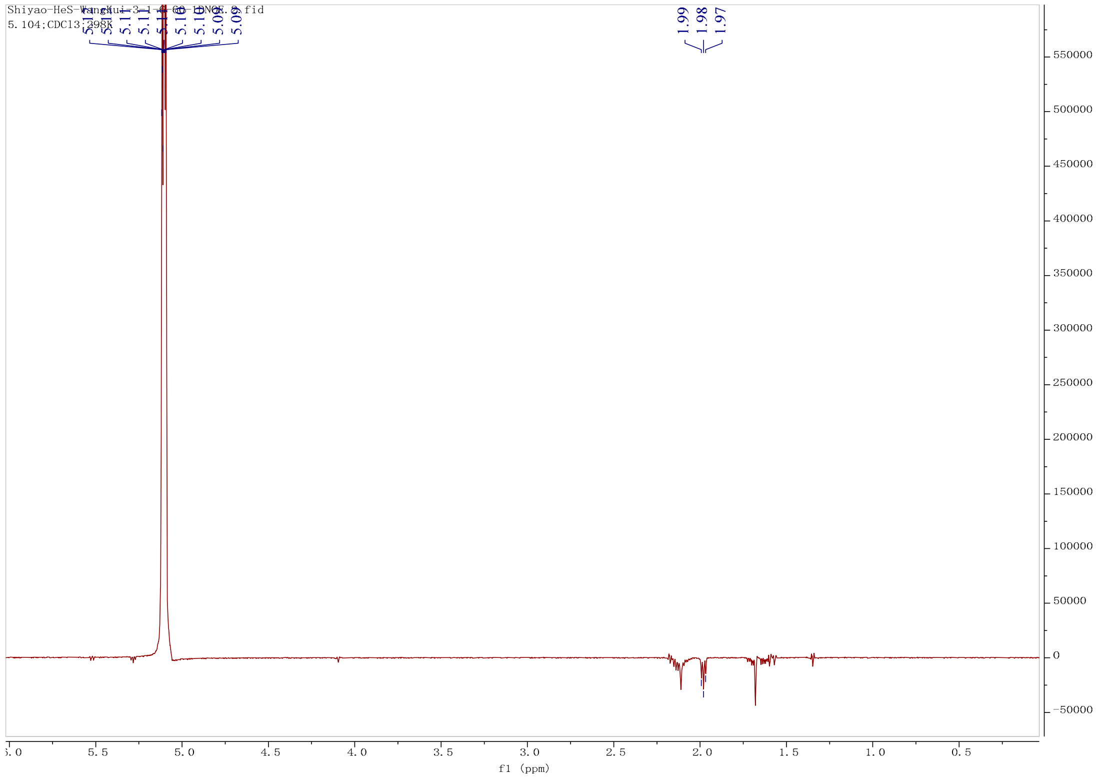

Figure S16 1D NOE spectrum of **2** in $CDCl_3$, 600 MHz (H-6)

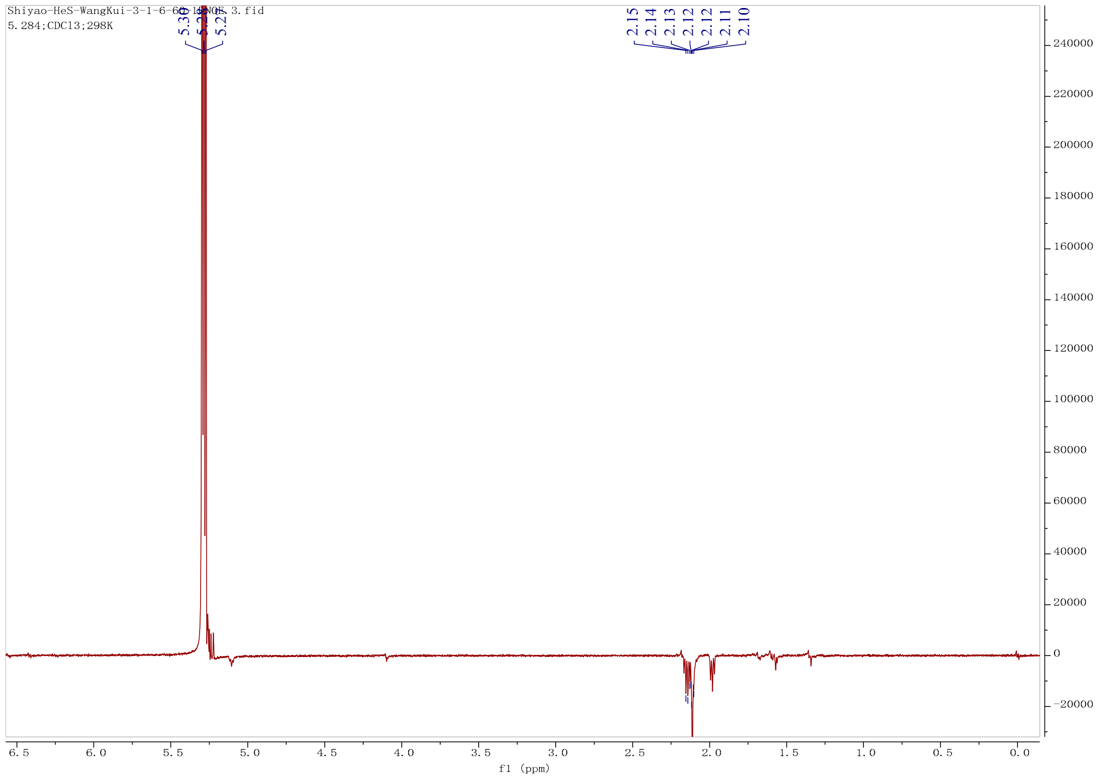

Figure S17 1D NOE spectrum of **2** in $CDCl_3$, 600 MHz (H-10)

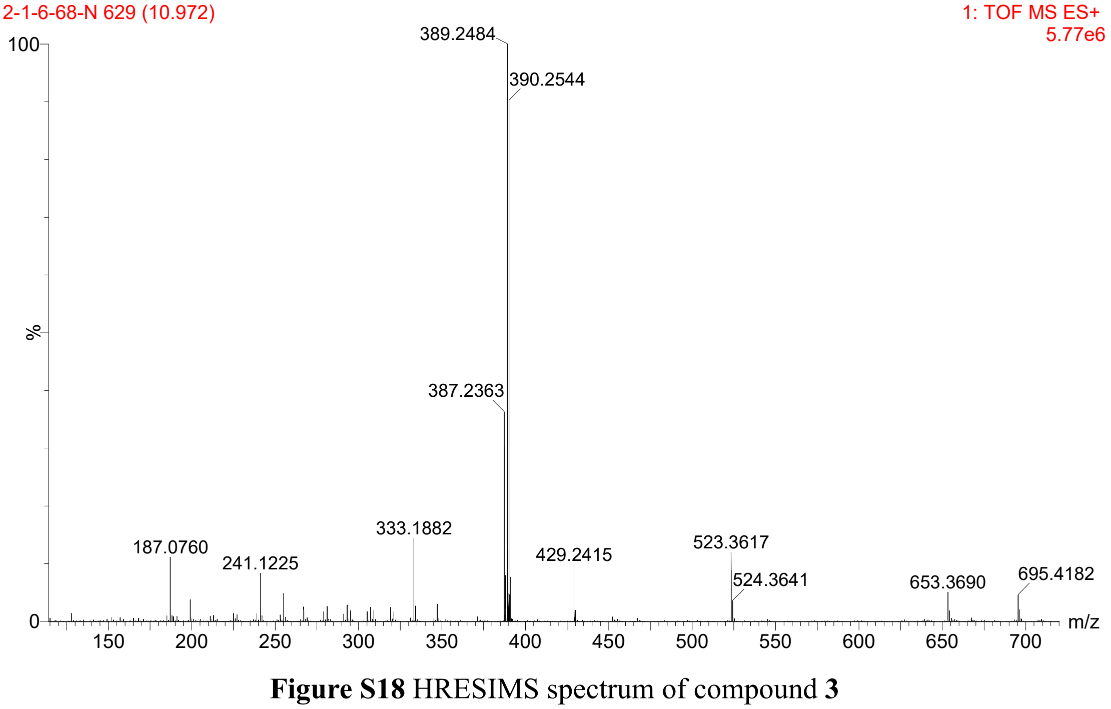

Figure S18 HRESIMS spectrum of compound **3**

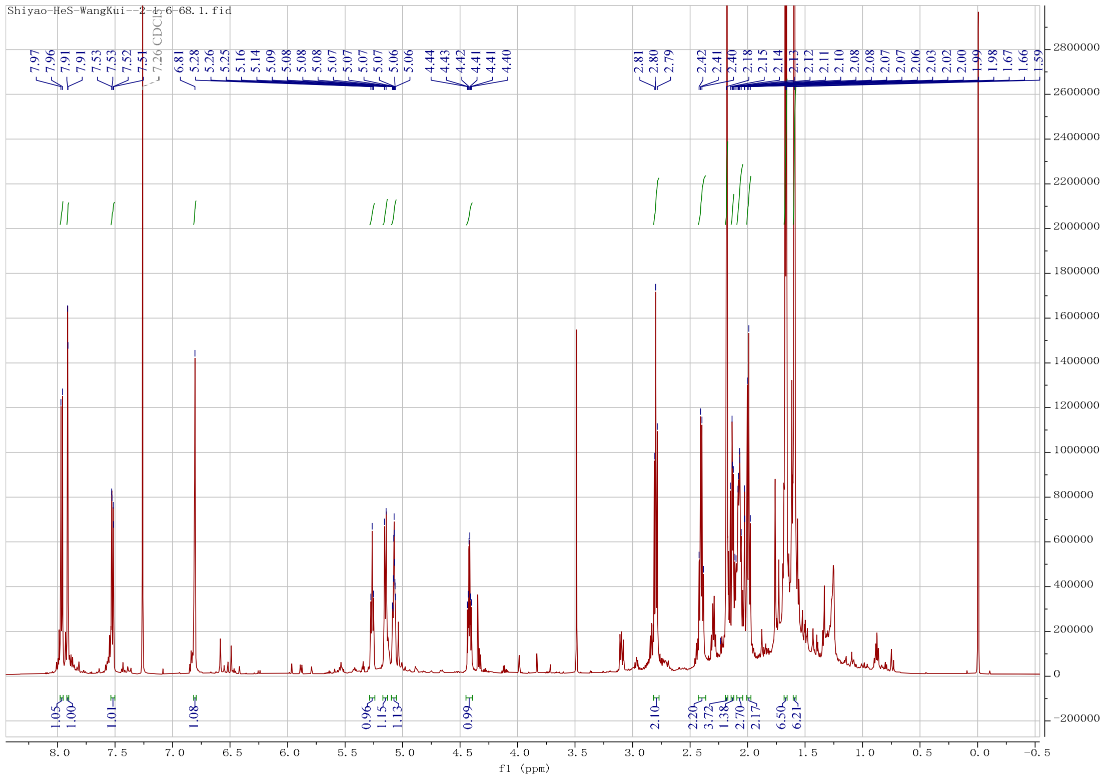

Figure S19 ^{1}H NMR spectrum of compound **3** in $CDCl_3$, 600 MHz

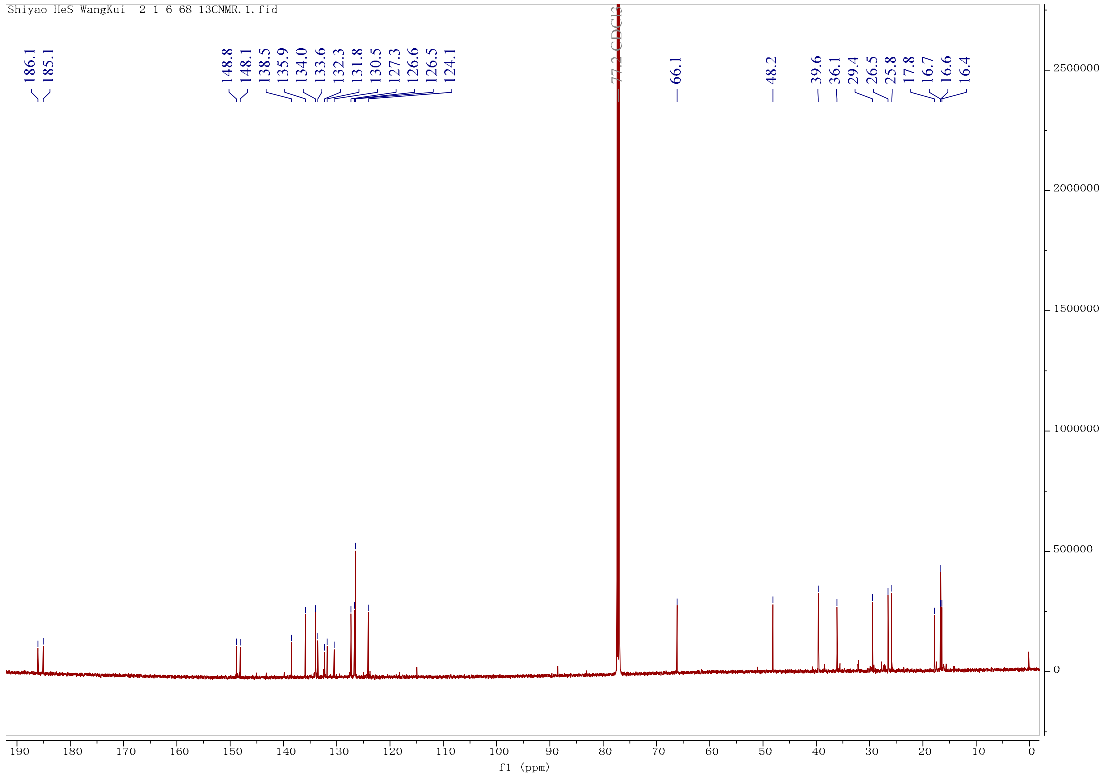

Figure S20 ^{13}C NMR spectrum of compound **3** in $CDCl_3$, 150 MHz

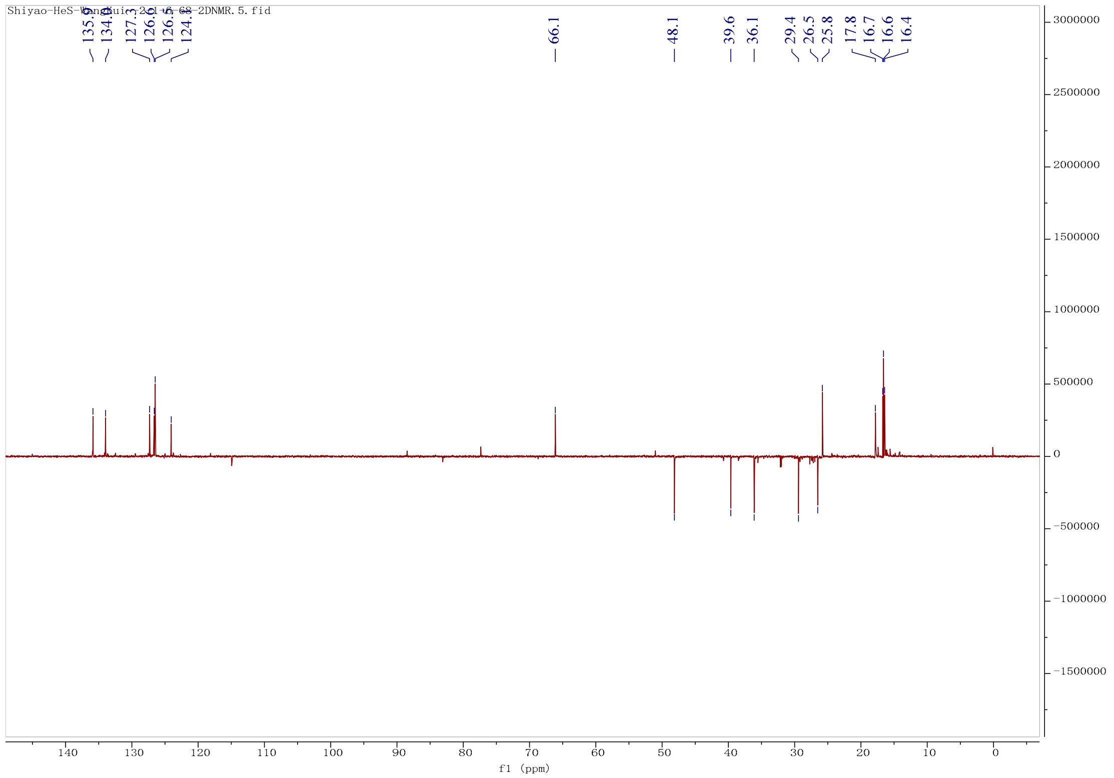

Figure S21 DEPT 135 spectrum of **3** in $CDCl_3$, 150 MHz

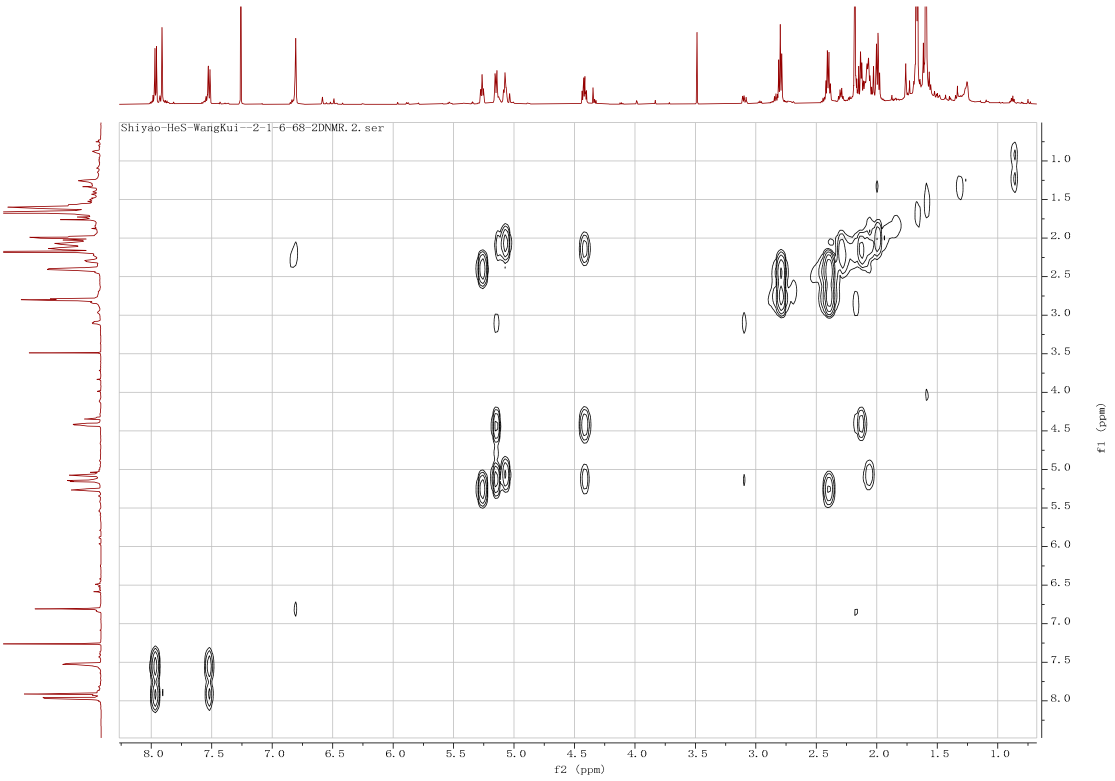

Figure S22 ^1H-^1H COSY spectrum of **3** in $CDCl_3$, 600 MHz

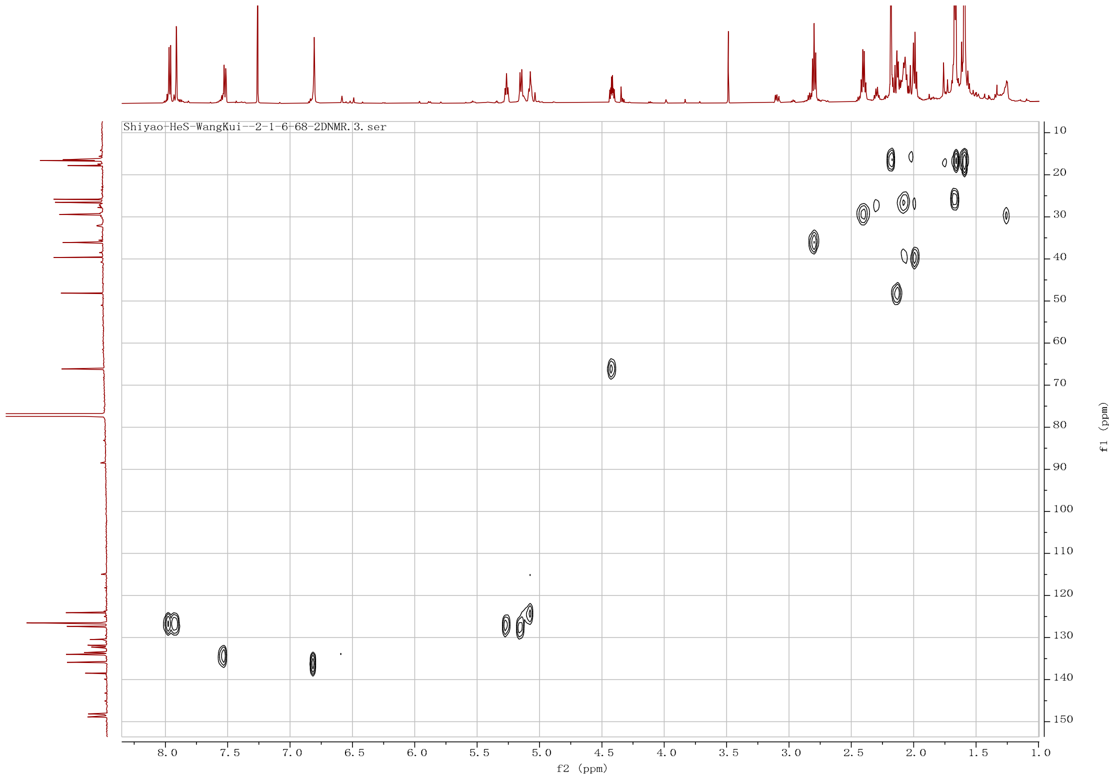

Figure S23 HSQC spectrum of **3** in $CDCl_3$, 150 MHz

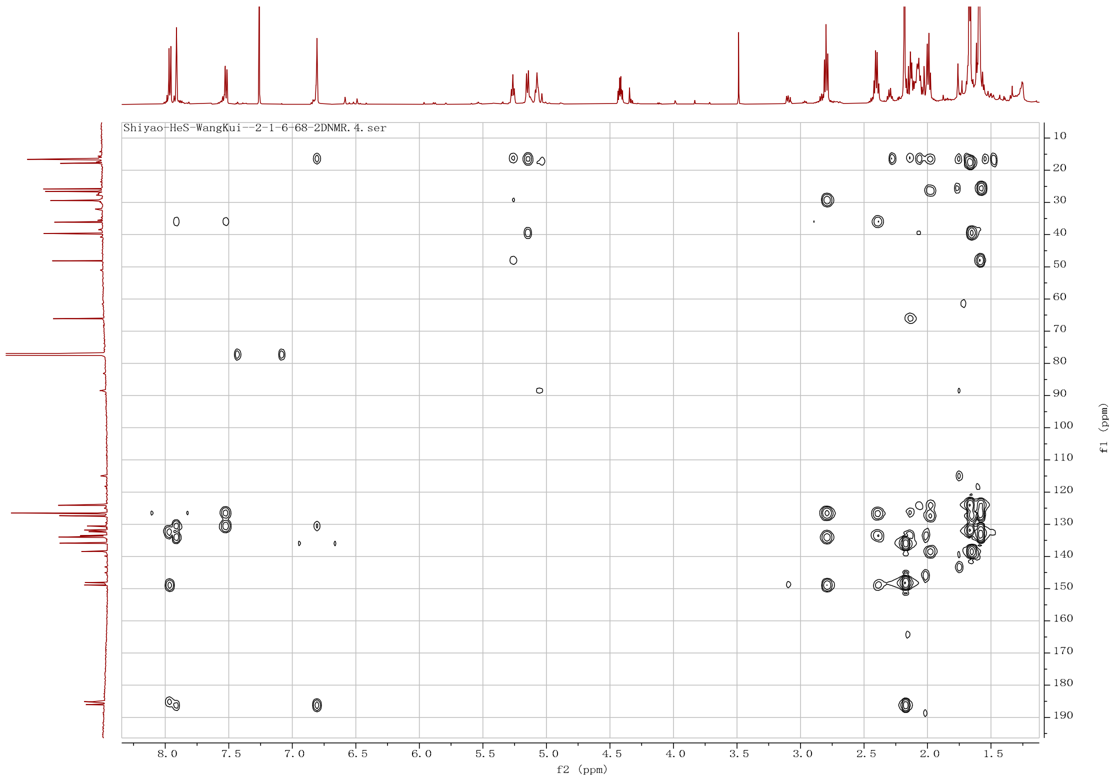

Figure S24 HMBC spectrum of **3** in $CDCl_3$, 150 MHz

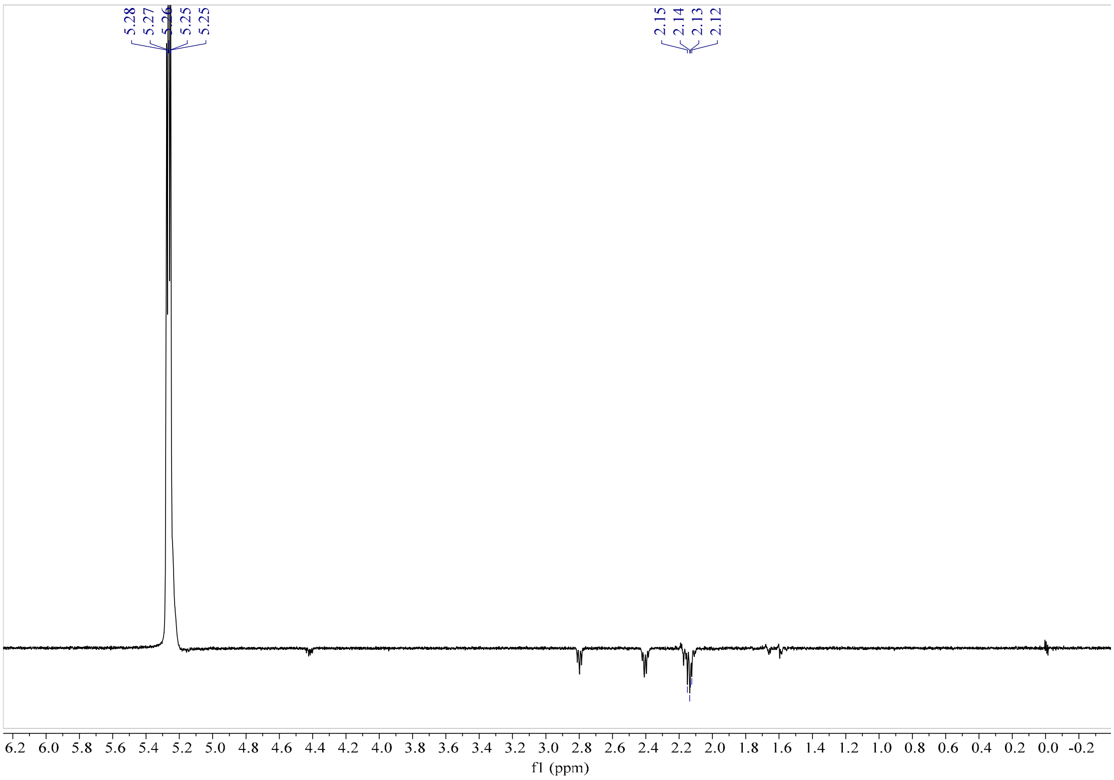

Figure S25 1D NOE spectrum of **3** in $CDCl_3$, 600 MHz (H-6)

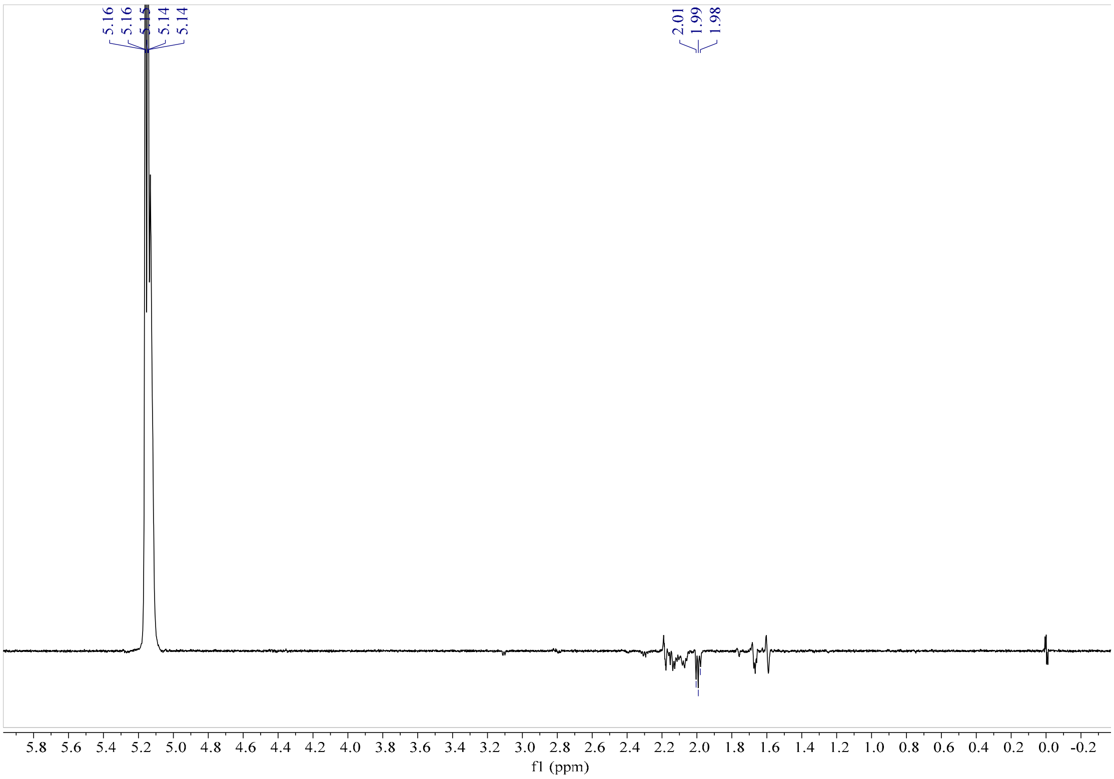

Figure S26 1D NOE spectrum of **3** in $CDCl_3$, 600 MHz (H-10)

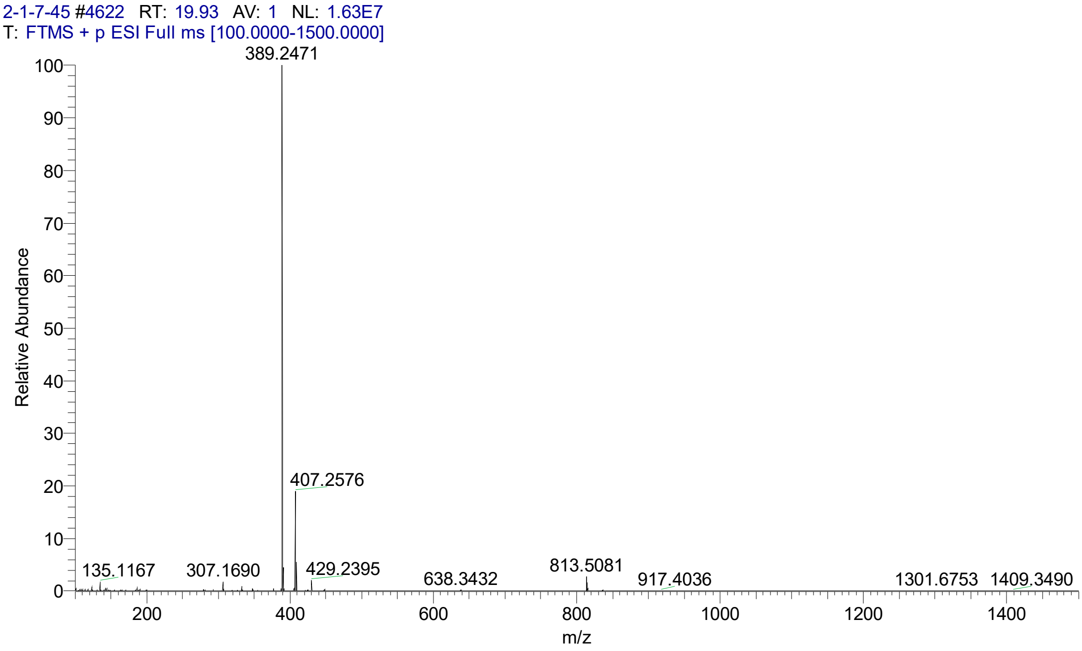

Figure S27 HRESIMS spectrum of compound **4**

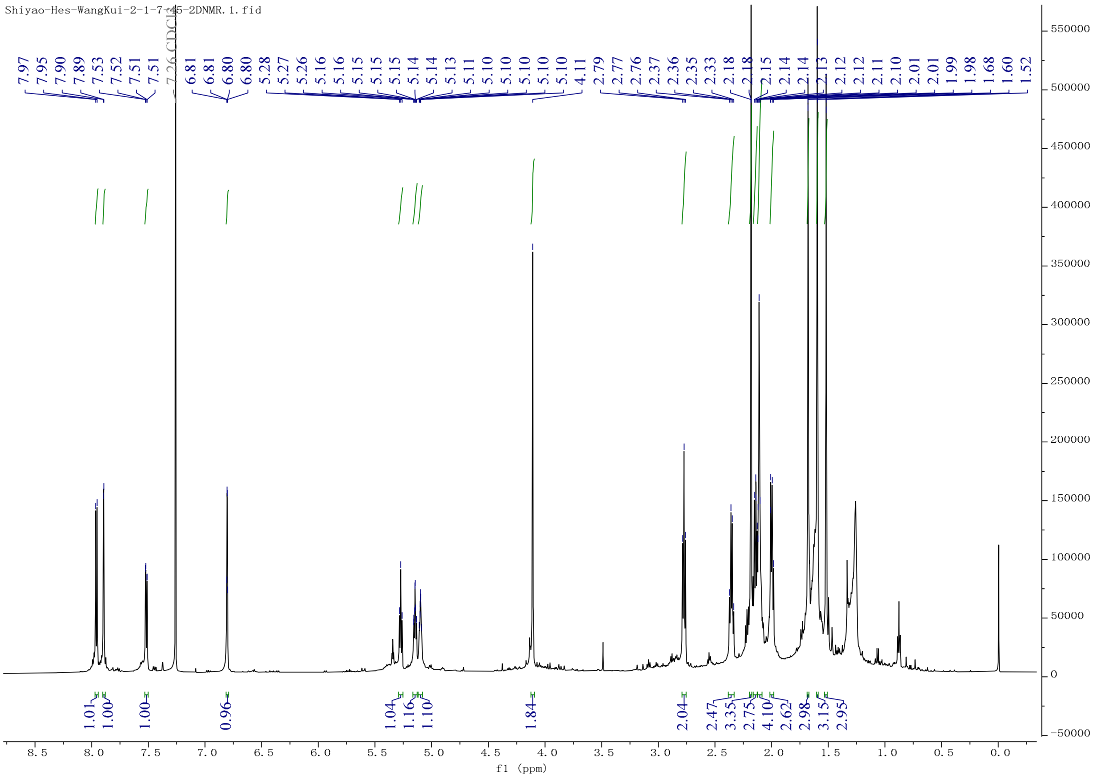

Figure S28 ^{1}H NMR spectrum of compound **4** in $CDCl_3$, 600 MHz

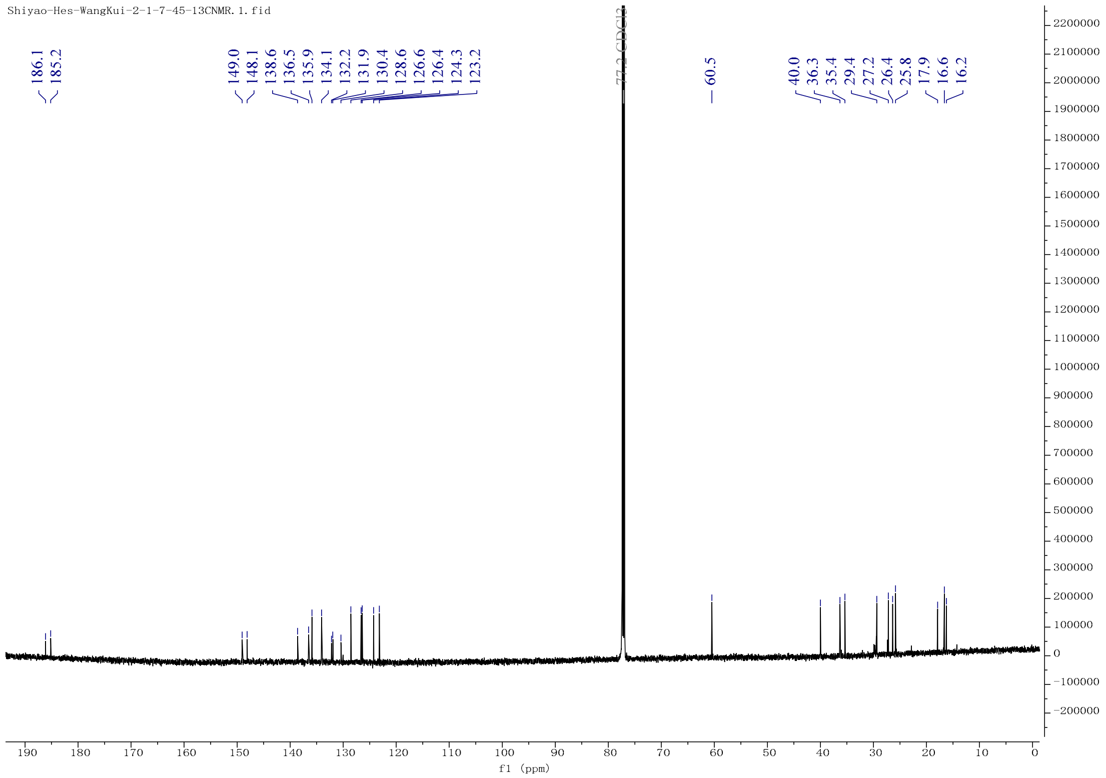

Figure S29 ^{13}C NMR spectrum of compound **4** in $CDCl_3$, 150 MHz

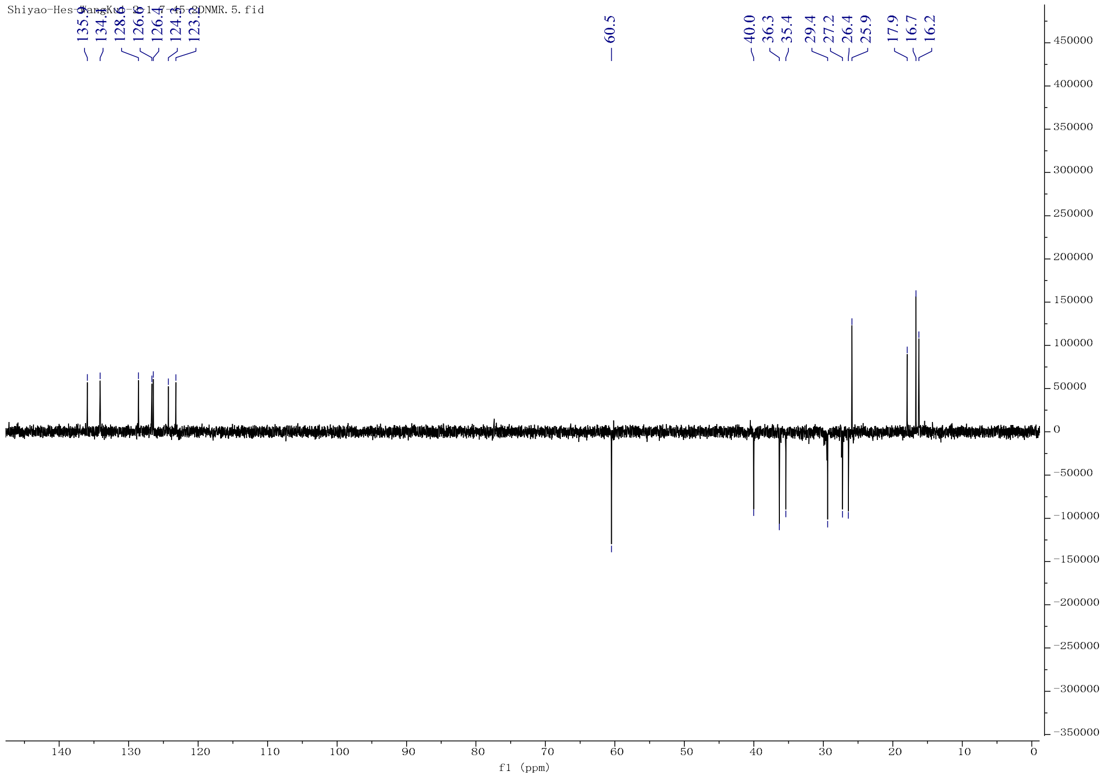

Figure S30 DEPT 135 spectrum of **4** in $CDCl_3$, 150 MHz

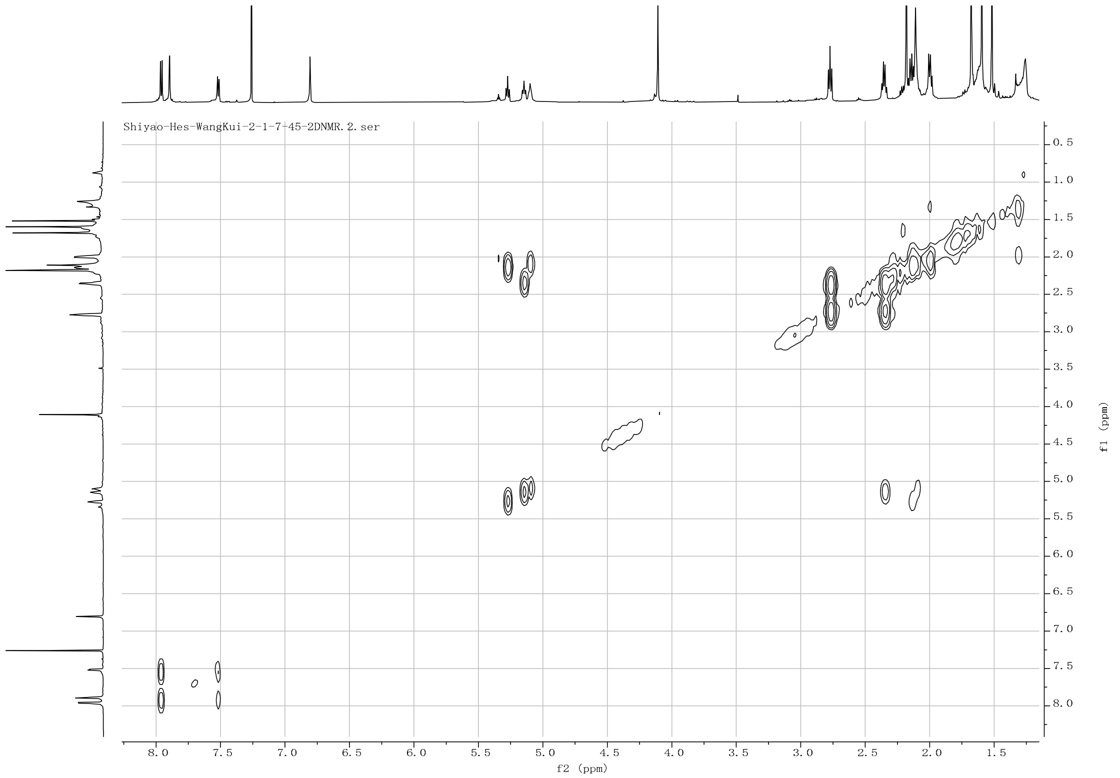

Figure S31 ^{1}H-^{1}H COSY spectrum of **4** in $CDCl_3$, 600 MHz

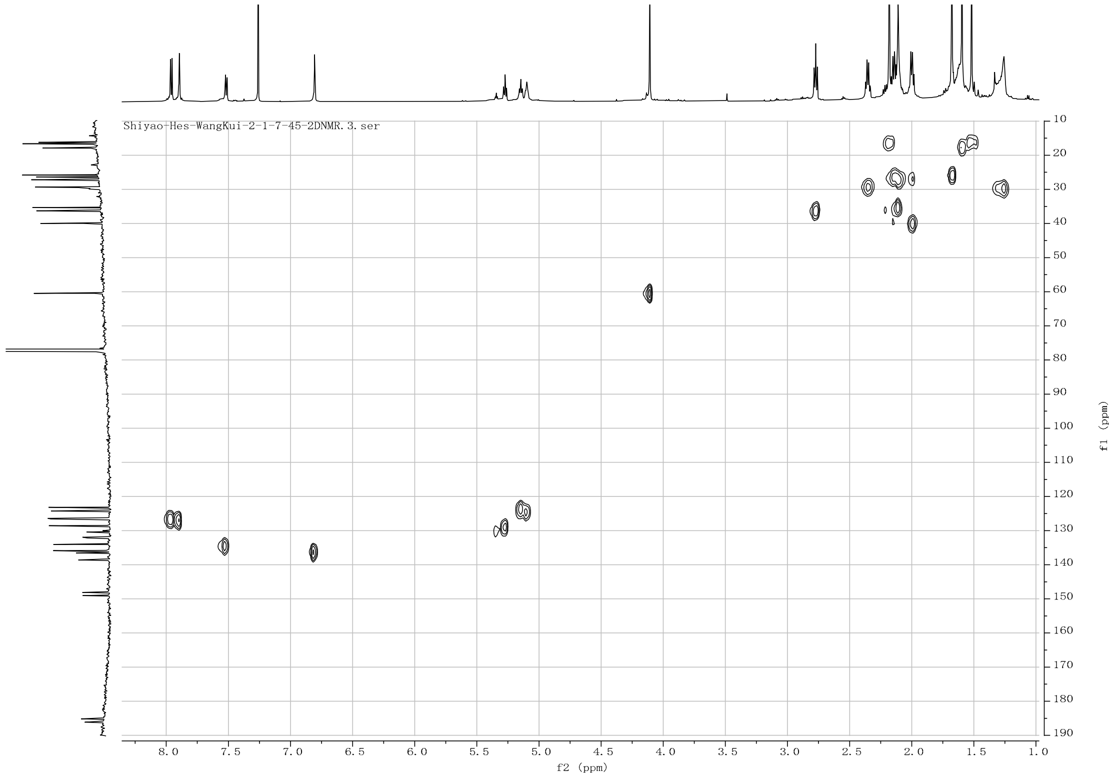

Figure S32 HSQC spectrum of **4** in $CDCl_3$, 150 MHz

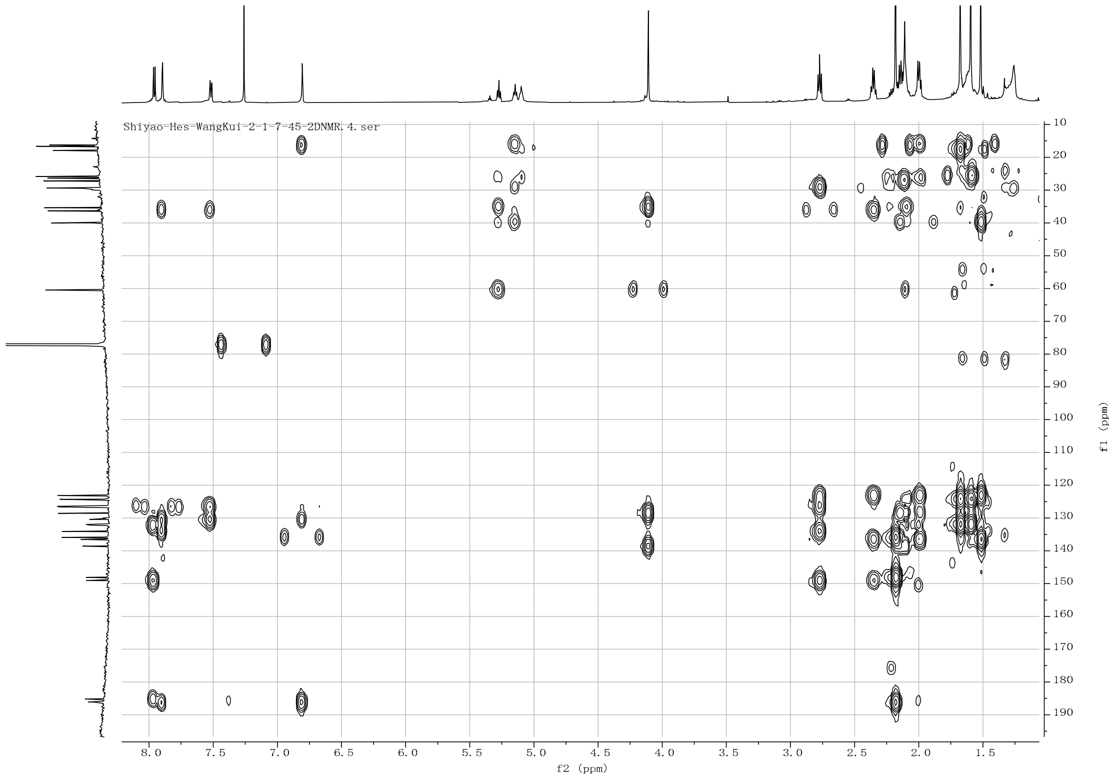

Figure S33 HMBC spectrum of **4** in $CDCl_3$, 150 MHz

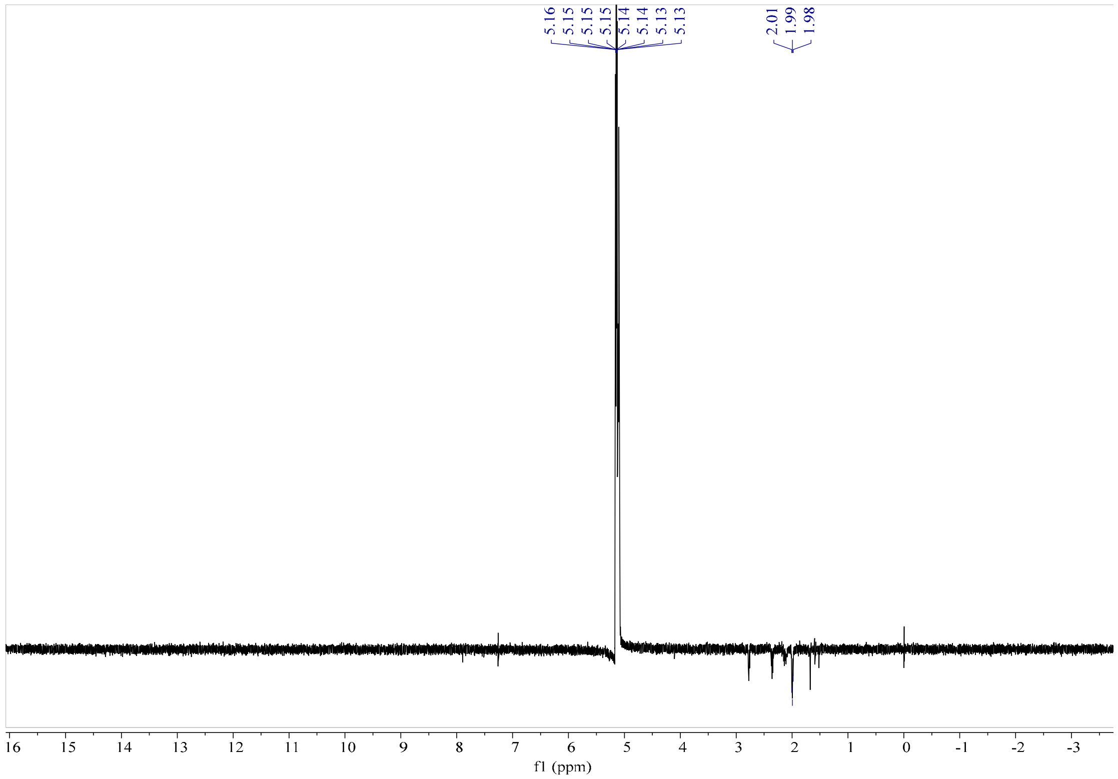

Figure S34 1D NOE spectrum of **4** in $CDCl_3$, 600 MHz (H-6)

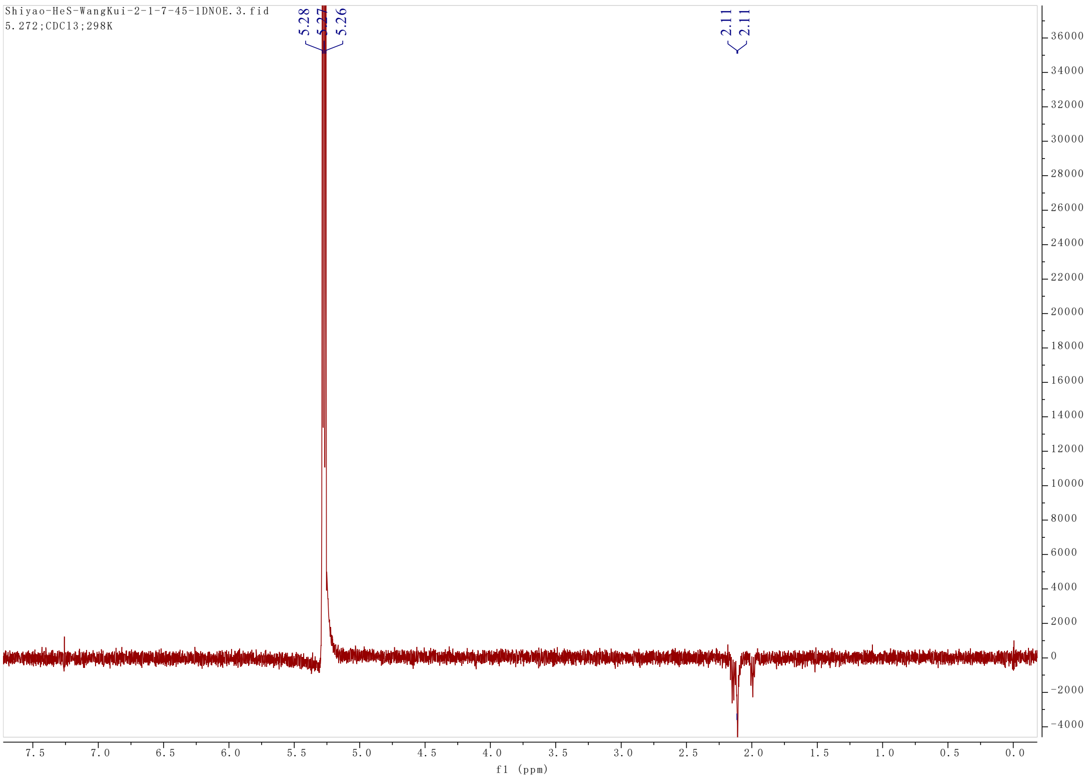

Figure S35 1D NOE spectrum of **4** in $CDCl_3$, 600 MHz (H-10)

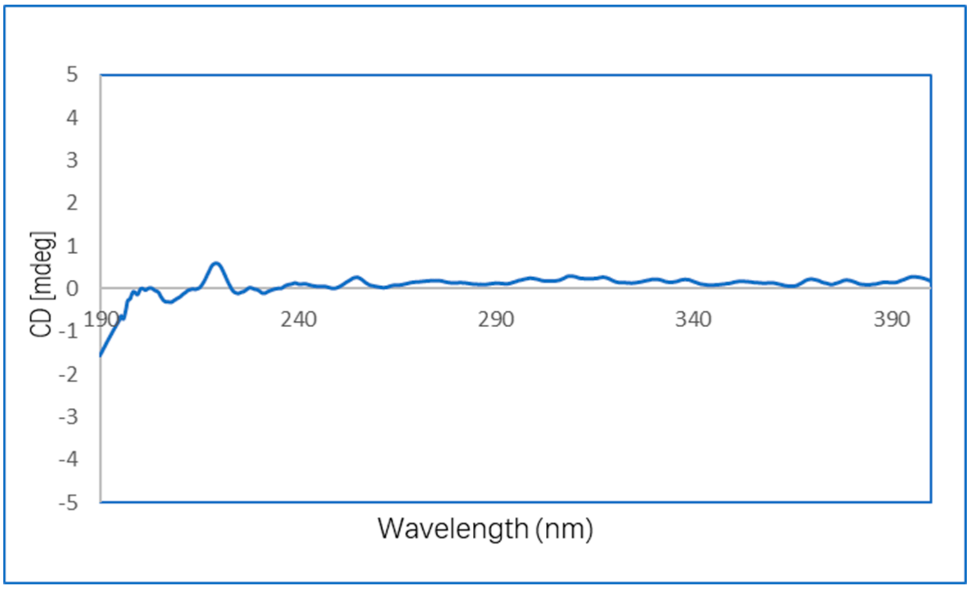

Figure S36 The experimental ECD spectrum of **1**

Figure S37 The experimental ECD spectrum of **2**
